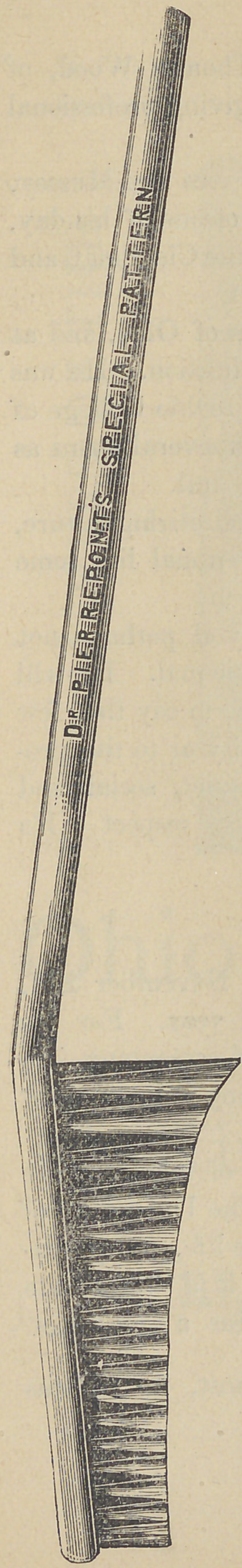# A New “Special” Pattern Tooth Brush

**Published:** 1880-12

**Authors:** 


### A NEW "SPECIAL" PATTERN TOOTH BRUSH.

Reference was made in the REGISTER some months ago to the Tooth-Brush here illustrated. I call attention to it here again, because the cut is now at hand, and gives a more perfect idea than can be derived from a mere description.

I have had this form of brush in use for about one year, and it is certainly the best with which I am familiar. Dr. Pierrepont has certainly, by this device, conferred a great benefit upon the profession and upon the public.

The House of S. S. White, of Philadelphia, is the agent in this country. They can be procured through any Dental depot.

## Figures and Tables

**Figure f1:**